# Direct monitoring of bias-dependent variations in the exciton formation ratio of working organic light emitting diodes

**DOI:** 10.1038/srep15533

**Published:** 2015-10-21

**Authors:** Takahiro Takahashi, Katsuichi Kanemoto, Mariko Kanenobu, Yuta Okawauchi, Hideki Hashimoto

**Affiliations:** 1 Department of Physics, Osaka City University, 3-3-138 Sugimoto, Sumiyoshi-ku, Osaka 558-8585, Japan; 2 The OCU Advanced Research Institute for Natural Science and Technology (OCARINA), Osaka City University, Osaka 558-8585, Japan

## Abstract

In typical operation of organic light emitting diodes (OLEDs), excitons are assumed to generate with a ratio of 1:3 for singlet and triplet excitons, respectively, based on a simple spin statistics model. This assumption has been used in designing efficient OLEDs. Despite the larger generation ratio of triplet excitons, physical properties of fluorescent OLEDs are usually evaluated only through the electroluminescence (EL) intensity from singlets and the behaviors of triplets during the LED operation are virtually black-boxed, because the triplets are mostly non-emissive. Here, we employ transient spectroscopy combined with LED-operation for directly monitoring the non-emissive triplets of working OLEDs. The spectroscopic techniques are performed simultaneously with EL- and current measurements under various operation biases. The simultaneous measurements reveal that the relative formation ratio of singlet-to-triplet excitons dramatically changes with the magnitude of bias. The measurements also show that the generation efficiency of singlets scales with the bias, whereas that of triplets is nearly bias-independent. These features of the formation ratio and efficiency are compatibly explained by considering the yield of intersystem crossing and the energy separation of excitons from electron-hole pairs. The obtained findings via the spectroscopic measurements enable prediction of the formation pathways in OLEDs.

In recent years, because of the outstanding progress in organic light emitting diodes (OLEDs), there have been growing interests in OLEDs toward next-generation displays and illumination light sources. In typical operation of OLEDs, excitons are assumed to generate with a ratio of 1:3 for singlet and triplet excitons (SEs and TEs), respectively, according to simple spin statistics^1^. The internal quantum efficiency of fluorescent OLEDs is then limited to 25% at its maximum by the statistics. However, recent studies suggested that the generation fraction of SEs significantly exceeds the statistical limit by the use of delayed fluorescence resulting from triplet-triplet annihilation (TTA)^2^,^3^,^4^,^5^ and thermally activated delayed fluorescence^6^,^7^. Utilizing such secondary formation processes of excitons is the recent key strategy for designing highly efficient OLEDs, and it requires an explicit understanding of the formation process.

Including the cases of the delayed luminescence, all types of OLEDs operate via the recombination of injected positive and negative carriers (polarons) and subsequent formation of weakly bound electron-hole pairs (polaron pairs: PPs). The PPs branch into singlet and triplet PPs (SPPs and TPPs) with a ratio of 1:3 and the spin statistics model expects SEs and TEs to take over the branching ratio^1^. However, several reports suggested that the formation ratio of TE to SE, *γ*, could deviate from 3^8^,^9^,^10^,^11^,^12^,^13^,^14^,^15^,^16^. Moreover, *γ* was suggested to change depending on the operation bias of OLEDs^11^,^13^, although the effect of bias on the exciton formation processes is usually not considered in the spin statistics model. The exciton formation processes thus still remain unclear despite being essential to every OLED, and resolving such issues of the formation processes is required for elaborate design of efficient OLEDs.

In general, the operation process of fluorescent OLEDs is evaluated only through the behaviors of SE monitored from the electroluminescence (EL) intensity, because TEs are mostly non-emissive. Therefore, establishing a reliable monitoring method for TEs is crucial for explicit evaluation of the formation process. Spectroscopic techniques are then straightforward for TE-detection and several groups indeed employed the techniques to observe TEs generated in OLEDs^12^,^13^,^16^,^17^. Lin *et al.* suggested that *γ* could be significantly reduced with the increase of an electric field from biases^13^. However, their results were questioned because of unresolved spectroscopic signals that could include contributions from carriers^18^,^19^. Walikewitz *et al.* reported detailed spectroscopic studies on the dynamics of TEs in polymer LEDs^17^. They demonstrated that the TE-dynamics are dominated by subsequent TTA. However, alternatively, the *intrinsic* formation processes of excitons from PPs were concealed by the TTA and not observed. Therefore, employing sensitive spectroscopic techniques and relevant material systems is required for precisely evaluating the intrinsic processes of excitons.

In this article, the dynamical processes of TEs during LED operations are directly investigated by spectroscopic techniques combined with operation of fluorescent polymer light emitting diodes (PLEDs). Transient spectroscopy measurements were employed and simultaneously performed with EL measurements, enabling simultaneous monitoring of time-evolution in SEs and TEs during the EL operation. Poly[2-methoxy-5-(2-ethylhexyloxy)-1,4-phenylenvinylen] (MEH-PPV), one of the representative fluorescent polymers, was employed as the emission layer of the PLED. The use of this polymer was designed to precisely evaluate the intrinsic exciton generation process, because this polymer exhibits no delayed luminescence as shown hereafter. The simultaneous measurements were performed in a wide bias range. As a consequence, the formation ratio of SE to TE is shown to scale dramatically with the operating bias. Simultaneous current measurements further demonstrate that the generation efficiency of SE increases with the bias, whereas that of TE is nearly bias-independent. The reason of the bias-enhanced SE-generation is discussed and fully explained by considering the larger energy separation of SE from PPs than that of TE. These findings confirm that the excitons do not simply take over the formation ratio of PPs, and we emphasize the necessity of considering the bias effect for designing efficient OLEDs.

## Results

**Figure 1:**
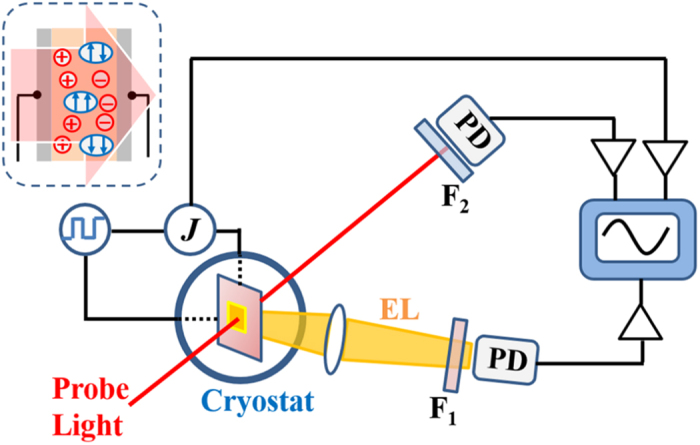
Schematics of experimental setup used for simultaneous measurements. A modulated square-wave bias is applied to a polymer LED set inside an evacuated cryostat. Modulated signals from the probe light and EL for detecting triplet and singlet excitons, respectively, are simultaneously recorded with a digital oscilloscope. Optical filters, *F*_1_ and *F*_2_ were used to block scattered probe light and EL, respectively. Current induced by the bias was also simultaneously recorded.

**Figure 2:**
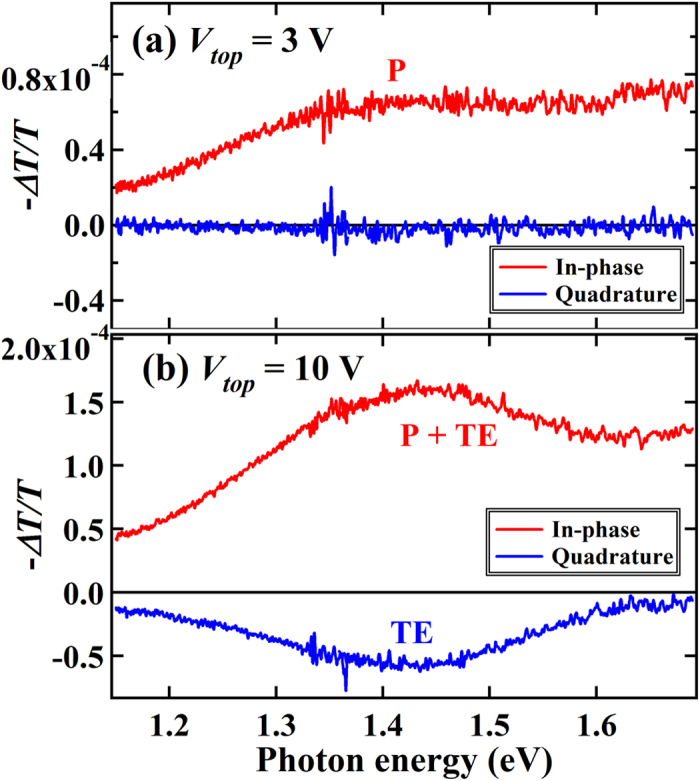
Spectra obtained from bias-modulation (BM) measurements. (**a**) Spectra under the bias voltage (*V*_top_) of 3 V and (**b**) of 10 V for the MEH-PPV diode. The components of in-phase and quadrature phases are obtained from dual-phase lock-in techniques.

The spectroscopic measurements combined with the LED-operation were performed by applying a modulated square-wave bias to the LED and detecting the modulation signals of the transmitted probe light, hereafter termed bias-modulation (BM) spectroscopy (Fig. 1)^12^,^20^,^21^. Figure 2(a,b) show the BM-spectra for the MEH-PPV device measured under the bias voltages of 3 V and 10 V, respectively. Both spectra were recorded with a dual phase lock-in technique sensitive to discriminating components differing in the response rate for modulation. The spectra under 3 V consist entirely of only the in-phase component with an absorption peak at approximately 1.4 eV. The absorption peak is close to the polaron peak at 1.35 eV determined previously from absorption-detected magnetic resonance (ADMR) measurements for MEH-PPV films^22^, and thus attributed to the injected polarons that serve as trapped or mobile carriers. In contrast, the BM-spectra under 10 V consist of both the in-phase and quadrature components with a common peak around at 1.42 eV. The quadrature signals indicate appearance of signal components delayed to the squared modulation. Also the quadrature-spectrum is similar to the spectrum of TE identified from the ADMR measurements^22^. The signals are thus assigned to the T_1_-T_n_ transition (T_*i*_ is the *i*-th energy level of TE) of the TE generated by the LED operation. Although Lin *et al.* suggested a similar assignment to the absorption signals at 1.4 eV as belonging to the TE transition^13^, the assignment was questioned because the signals may include the polaron transitions^18^,^19^. Indeed, the in-phase signals at 1.42 eV under 10 V consist of both the polaron and TE signals.

**Figure 3:**
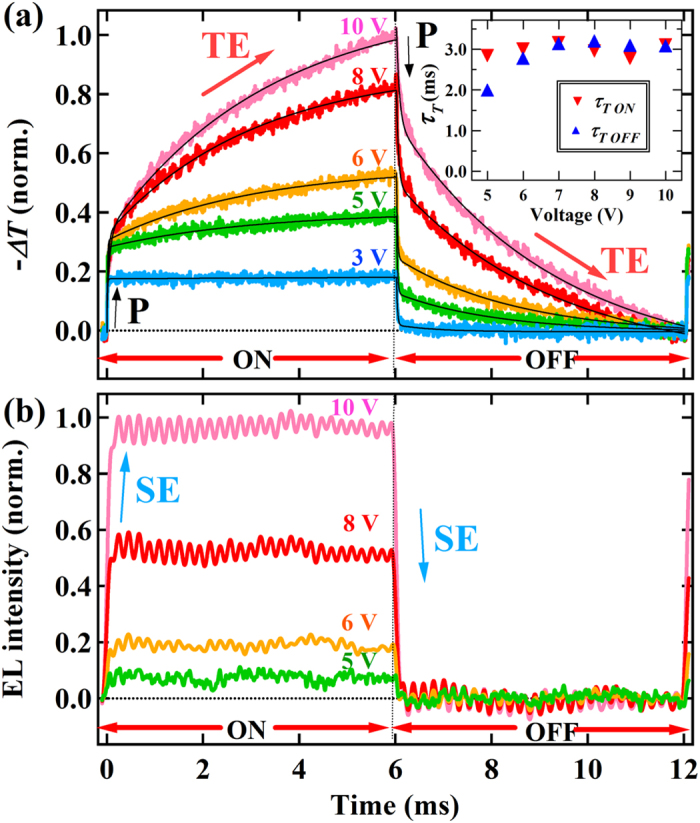
Time profile of simultaneous transient BM and EL measurements when turning on and off the LED. (**a**) Transient-BM and (**b**) -EL signals measured for the MEH-PPV LED under a top voltage range from 3 to 10 V. The solid curves in (**a**) are the results of fit using two exponential functions. The insets of (**a**) show the lifetimes of triplet exciton (TE) during LED-on and -off (*τ*_*T,ON*_ and *τ*_*T,OFF*_, respectively) determined from the exponential fits.

Figure 3(a,b) present the results of the simultaneous measurements of transient BM at 1.42 eV and EL under the voltage range of 3 to 10 V, which visualize the generation and decay processes of TEs and SEs, respectively. The BM signals at 1.42 eV have two components: rapid and slow components that rise and decay within 10 μs and on millisecond timescales, respectively. The slow component was observed only at the bias voltages beyond 3 V, which coincided with the voltage where EL was detected. This observation and the spectral features described above confirm that the rapid and slow components of the BM signals correspond to the signals of polarons and TEs, respectively. Unlike the case of the BM signals, the EL signals rapidly rise and decay within 10 μs and do not contain slow components. In the case of fluorescent EL resulting from TTA, the turn-off transients of EL exhibit a delayed fluorescence related to the TE-lifetime^2^,^17^. The absence of such delayed EL signals in Fig. 3(b) demonstrates that the effect of TTA is negligible in the MEH-PPV LED. Therefore the use of this polymer LED enables studies of the intrinsic exciton formation processes without overcoming the difficulty in resolving contributions of subsequent exciton effects.

By neglecting the TTA process, the generation process of TE is simplified by the following rate equation:


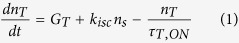


where *n*_S_ and *n*_T_ are the densities of SE and TE, *G*_*T*_ and *k*_isc_ are the generation rate of TE from TPPs and the intersystem crossing (ISC) rate from SEs, respectively, and *τ*_*T,ON*_ the TE-lifetime during the LED operation. In the timescale of the TE-lifetime, the initial rise of *G*_*T*_ is very fast and *n*_S_ immediately reaches steady state corresponding to *G*_*S*_ *τ*_*S*_, with *G*_*S*_ and *τ*_*S*_ being the generation rate of SE from SPPs and the SE-lifetime, respectively. The time evolution of *n*_T_ is then obtained as follows, by assuming the sum *G*_*T*_ + *k*_isc_ *G*_*S*_ *τ*_*S*_ (=*G*′_*T*_) to be time-independent:


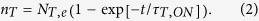


Here *N*_*T,e*_ is the TE-density in the equilibrium state corresponding to the product *G*′_*T*_*τ*_*T,ON*_. The time response of polarons is fast and could be beyond the time resolution of the experimental system in this study. However, by approximating the rise process of polarons with a single exponential function, the overall rise process of the transient BM signals was fitted by the sum of the two exponential functions. The overall decay process was also fitted by the sum of exponential decay functions with *τ*_*T,OFF*_ being the TE-lifetime under off-bias of the LED. The fit-results shown in Fig. 3(a) reproduce well both the rise and decay processes of BM signals and can be used to determine *τ*_*T,ON*_, *τ*_*T,OFF*_ and the relative values of *N*_*T,e*_. The details of the obtained fitting parameters are shown in Supplementary Information.

The inset of Fig. 3(a) shows that both *τ*_*T,ON*_ and *τ*_*T,OFF*_ only weakly depend on voltage despite obvious increases of the TE-density by the voltage. This feature indicates that the effect of the TE-TE interaction is negligible, being consistent with the result of EL that exhibited no delayed components resulting from TTA. The inset also shows that the difference in the lifetimes is very small. Generally, when there are frequent TE-polaron collisions^23^, they should give rise to a difference between *τ*_*T,ON*_ and *τ*_*T,OFF*_ because the polaron density at the on-bias is much larger. Therefore, the influence of the TE-polaron interaction on the TE-dynamics is negligible in the MEH-PPV LED although the rapid quenching of TE was reported on other polymer LEDs^24^. Unlike the case in TE, *τ*_*S*_ cannot be determined from the transient EL because *τ*_*S*_, typically on the order of 0.1 ~ 1 ns^25^,^26^,^27^, is much smaller than the circuit time constant of the experimental system (~1 μs). However, we can expect SEs to have smaller probability to encounter polarons than TEs because of their much shorter lifetimes. Therefore, the influence of the SE-polaron interaction on the SE-dynamics should be negligible and *τ*_*S*_ is regarded as nearly voltage-independent.

**Figure 4:**
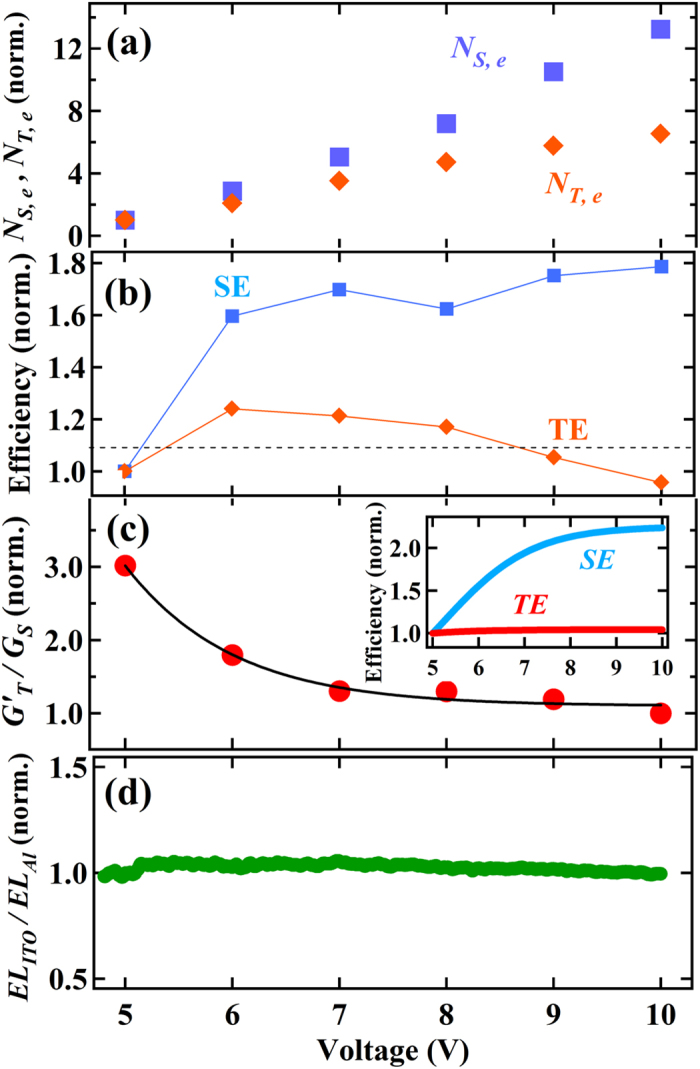
Bias-dependent shifts of important parameters. (**a**) Equilibrium densities of singlet exciton (SE) (*N*_*S,e*_) and triplet exciton (TE) (*N*_*T,e*_) normalized at 5 V. (**b**) Conversion-efficiencies normalized at 5 V of SE and TE calculated by the EL- and BM-intensity divided by current measured simultaneously vs. applied voltages. The horizontal dotted line indicates the mean value for TE. (**c**) Relative ratio of the generation rate of SE to that of TE normalized at 10 V versus applied voltage. The solid curve indicates the best-fit result according to equation (4). The inset indicates the generation efficiencies of SE and TE vs. voltage obtained from the best-fit result of (**c**). (**d**) Ratio of the EL-intensity measured through the anode (ITO) to that through the cathode (Ca:Al) normalized at 10 V. The EL intensities were simultaneously measured using two photo-diodes set facing the anode and the cathode sides.

Figure 4(a) plots the voltage-dependence of the relative *N*_*S*_,_e_ and *N*_*T,e*_ (*N*_*S,e*_ is the equilibrium SE-density) obtained from the results of Fig. 3. This plot indicates that the ratio of the exciton density significantly changes depending on the operating bias, and particularly that the fraction of SE scales with the bias. In order to elucidate factors determining the bias dependence, the relative quantum efficiencies of SE and TE were calculated from *N*_*S*,e_ and *N*_*T,e*_ divided by the saturation current measured simultaneously with the transient BM and EL measurements. The results shown in Fig. 4(b) demonstrate that the efficiency of SE scales with the operating bias, whereas that of TE is nearly bias-independent. It is thus suggested that the observed voltage-dependent ratio of exciton density is caused by properties of SEs.

We herein evaluate the voltage-dependence of *G*_*T*_/*G*_*S*_, corresponding to *γ*, for explaining the voltage-dependent *N*_*S*_,_e_ and *N*_*T,e*_. The relative intensity of *G*′_*T*_/*G*_*S*_ was thus calculated by neglecting the voltage-dependence of *τ*_*S*_ based on the discussion above. The result plotted in Fig. 4(c) shows that *G*′_*T*_/*G*_*S*_ is appreciably reduced by increasing the bias. The relation between *G*′_*T*_/*G*_*S*_ and *G*_*T*_/*G*_*S*_ is obtained by


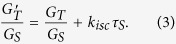


It was previously identified that the photoluminescence (PL) intensity of MEH-PPV weakly depends on the reverse electric field and that the change of PL intensity is explained only by considering the dissociation of excitons into carriers^28^. This indicates that the voltage dependence of the ISC process is negligible in MEH-PPV. Therefore, the voltage dependence of *G*′_*T*_/*G*_*S*_ in equation (3) is determined by the *G*_*T*_/*G*_*S*_ term, indicating that the result in Fig. 4(c) is caused by the trend of *G*_*T*_/*G*_*S*_ being reduced by increasing voltage. It is found that *G*′_*T*_/*G*_*S*_ is reduced to about one-third by changing the bias from 5 to 10 V. By using the photoluminescence-quantum efficiency of MEH-PPV being ~0.1^29^, the magnitude range of *G*_*T*_/*G*_*S*_ can be estimated from *G*′_*T*_/*G*_*S*_. For instance, when *G*_*T*_/*G*_*S*_ = 3 at 5 V, the estimated range is calculated as 0.4 ≤ *G*_*T*_/*G*_*S*_ ≤ 1 at 10 V. As discussed later, the ISC process in MEH-PPV is not efficient^30^, which makes *G*_*T*_/*G*_*S*_ close to the upper limit of the estimated range. In the model based on simple spin statistics, *G*_*T*_/*G*_*S*_ is assumed to take over the formation ratio of PPs (*γ* ~ 3) regardless of the operating bias. However, the estimated range in *G*_*T*_/*G*_*S*_ indicates that the excitons are unlikely to take over the spin statistics and that the operating bias significantly affects the exciton formation process.

## Discussion

We hereafter discuss the origin of the observed strong voltage-dependence of *G*_*T*_/*G*_*S*_. First, it was previously suggested that the excitons are quenched at the metallic cathode and the quenching effect could cause the voltage-dependent conversion-efficiency of PLED^31^. However, we note that such a quenching effect should be more noticeable in the longer-lived TE, which is inconsistent with the observed voltage-independent quantum efficiency of TE. Secondly, the PPs generated in the PLED can be dissociated by the external electric field and the dissociation could affect the voltage-dependence of *G*_*T*_/*G*_*S*_, because, if SPPs and TPPs are dissociated in different rates, it may lead to changing the density ratio of SE and TE. The dissociation-effect could particularly be important when the energy separation between SPPs and TPPs is not small. However, the energy separation was theoretically predicted to be very small^32^,^33^,^34^. Moreover, when such a dissociation effect is effective, the quantum efficiency of excitons should decrease with the bias, being inconsistent with the result for SEs in Fig. 4(b). Therefore the dissociation-effect is not the origin of the voltage-dependent *G*_*T*_/*G*_*S*_.

As further probable factors, the light-outcoupling efficiency (LOE) could affect the emission profile of PLEDs and cause voltage-dependent *G*_*T*_/*G*_*S*_^35^,^36^,^37^. Particularly, Carvelli *et al.* reported that, although the fraction of SE in PLEDs estimated from the EL-intensity changes depending on the applied voltage, the voltage-dependence can be explained by considering the LOE^35^. However, in the reference, the SE-fraction exhibited apparent decreasing trends with increasing voltage^35^, being opposite to the observation in this study. Also, such a voltage-dependent LOE is usually caused by the shift of emission zone toward the anode with increasing voltage. Therefore, if the LOE is strong, the ratio of EL-intensity measured through the anode and cathode should depend on voltage^36^. We examined the ratio for the present PLED and identified that it is nearly independent of voltage (Fig. 4(d)). Therefore, the LOE effect is unlikely to explain the observed voltage-dependent *G*_*T*_/*G*_*S*_.

**Figure 5:**
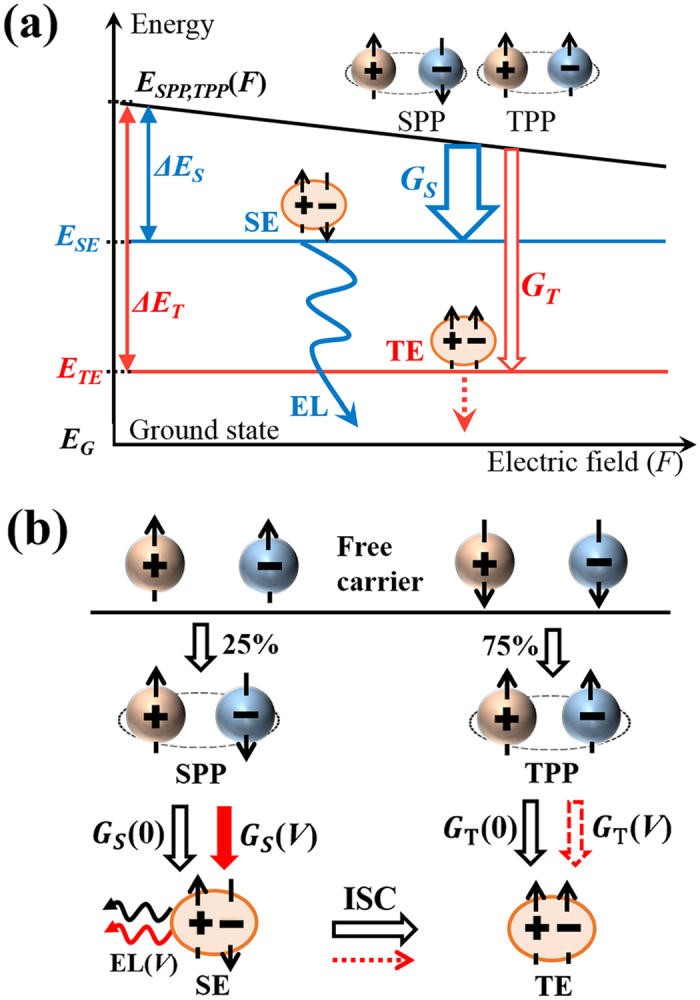
Schematics of bias-dependent formation pathways. (**a**) Schematic representation of the electric field *F*(*V*) dependence of the energy levels of SE (*E*_*SE*_), TE (*E*_*TE*_), singlet (triplet) polaron pair (PP) (*E*_*SPP(TPP)*_), and ground state (*E*_*G*_). *ΔE*_*S(T)*_ is the energy separation between SE (TE) and PPs. The energy levels of PPs decrease in proportion to the electric field and thereby the generation rate of SE (TE), *G*_*S*_(*G*_*T*_), depends on the field. (**b**) Bias-dependent formation pathways of excitons derived from the present study. The red solid (broken) arrows indicate the pathways strongly (weakly) depending on the bias. Remarkable bias effects only appear on emission from SE due to the inefficient intersystem crossing (ISC) yield in MEH-PPV.

From the discussions above, the voltage-dependent *G*_*T*_/*G*_*S*_ should be directly attributed to the generation process of excitons from PPs enhanced by an external electric field. A significant factor determining the generation rate is the energy separation between PPs and excitons^34^,^38^ denoted as *ΔE*_*S*_ and *ΔE*_*T*_ for SEs and TEs, respectively. When the dipole moment formed by the electron and hole of PPs interacts with an electric field, *ΔE*_*S*_ and *ΔE*_*T*_ are expected to decrease with increasing the field, because the longer electron-hole distance of PPs than excitons results in larger moments (Fig. 5(a)). Moreover, *ΔE*_*S*_ is typically much smaller than *ΔE*_*T*_ such that the energy difference between *ΔE*_*S*_ and *ΔE*_*T*_ is greater than 1 eV^39^. The smaller energy separation of *ΔE*_*S*_ results in larger enhancement of *G*_*s*_ than that of *G*_*T*_ in increasing the voltage and causes the decrease of *G*_*T*_/*G*_*S*_.

Related to this mechanism, Yin *et al.* calculated the dipole moment of PPs under an electric field for long-chain molecules and showed that the ratio of the formation cross section of TE to SE decreases with increasing an electric field^34^. We thus attempted to fit the results in Fig. 4(c) in reference to the calculation by Yin *et al.* using the following equation:


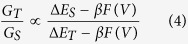


where *F*(*V*) is the electric field strength applied to PPs and *β* is the proportional constant commonly used for SPPs and TPPs. We then incorporated the screening effect by injected carriers for *F*(*V*) and assumed it to be saturated with increasing the bias according to the previous reports (Supplementary Information)^40^,^41^: we note that the saturation of the internal electric field was actually identified from spectroscopic measurements (published elsewhere). *ΔE*_*S*_ = 1.0 eV and *ΔE*_*T*_ = 2.2 eV were used based on the previous references^34^,^39^. The result of fit is shown in Fig. 4(c) and indicates that equation (4) successfully reproduces the voltage-dependent *G*′_*T*_/*G*_*S*_. However, we note that the voltage dependence of *G*′_*T*_/*G*_*S*_ was well-fitted only when neglecting the term of *k*_isc_ *τ*_s_ in equation (3), corresponding to *G*′_*T*_/*G*_*S*_ ~ *G*_*T*_/*G*_*S*_. This indicates that the formation process of TE via ISC is ineffective for this PLED, which is consistent with the previous report that showed the yield of ISC in MEH-PPV to be very small (~1%)^30^.

Considering equation (4), the transition probability from PPs to SE and TE is proportional to (*ΔE*_*S*_ − *βF*(*V*))^−1^ and (*ΔE*_*T*_ − *βF*(*V*))^−1^, respectively, and the relative value of each term can be calculated from the result of the model-fit. The inset of Fig. 4(c) shows the voltage-dependence of the calculated terms and indicates the trends of SE scaling with voltage and of TE being nearly voltage-independent. These calculated terms of transition probability are proportional to the relative quantum efficiency of excitons shown in Fig. 4(b) when ignoring the voltage dependence of the exciton lifetimes. Good agreement is found between the voltage dependency of the quantum efficiency and the transition probability. This agreement justifies the model proposed in this study and shows that the voltage-dependent *G*_*T*_/*G*_*S*_ is primarily determined by the *G*_*S*_-term increasing with voltage.

Figure 5(b) depicts the bias-dependent formation pathways of excitons derived from the present study. SPPs and TPPs generated at the ratio of 1:3 are converted into SEs and TEs while changing the generation ratio as a function of the bias. The voltage-enhanced generation of SE is responsible for the bias-effect, whereas the efficiency of the TE-generation from TPPs is nearly independent of bias. Although the voltage-enhanced SE-generation can also potentially enhance the generation efficiency of TE *via* the ISC process, such a pathway is neglected for the MEH-PPV LED because of its inefficient ISC yield. The resultant efficiency of the TE-generation thus remains bias-independent, being fairly consistent with the observations.

This mechanism was derived from the MEH-PPV LED and may not seem to be easily applied to other PLEDs. However the present research suggests that the exciton formation pathways of PLEDs should be virtually determined by two factors: the energy separation between *ΔE*_*S*_ and *ΔE*_*T*_ (~the energy separation of SE and TE) and the efficiency of ISC. Both factors have been examined and are known to differ depending on the molecular structure of polymers^30^,^39^,^42^. Considering these factors and subsequent exciton effects like TTA enables precise prediction of the bias-dependent operation process of PLEDs. We finally note that the bias-dependent changes in the energy separation between *ΔE*_*S*_ and *ΔE*_*T*_ were previously suggested to scale with a molecular length^34^. The degree of the bias-effect would thus be different between the cases of PLEDs and molecular-based OLEDs. Therefore careful re-evaluation is required for the degree of the bias-effect in the molecular OLEDs *via* finding appropriate model materials.

## Methods

### Sample Preparation

The polymer light emitting diodes used in this study were fabricated in the following sandwich structure: ITO/PEDOT-PSS/MEH-PPV/Ca/Al, where PEDOT-PSS is poly(3,4-ethylenedioxythiophene)-poly(styrenesulfonate). The MEH-PPV layer was produced in a glovebox by spin coating its chlorobenzene solution at a concentration of 5 mg/ml. The thickness of the polymer layer was about 50 nm. The Ca and Al layers at thicknesses of 5 nm and 20 nm, respectively, were deposited by vacuum evaporation and formed a semitransparent cathode. The size of active area was approximately 0.8 cm^2^.

### Spectroscopic measurements

The BM measurements were performed by applying a square-wave AC voltage to the PLEDs and detecting the modulation signals of the transmitted probe light synchronized with the frequency of the AC voltage (80 Hz). The probe light was produced using a tungsten/halogen lamp or LEDs. The spectra of BM signals were measured by using a phase-sensitive lock-in technique for the modulated probe light from the lamp passing through a monochromator. The probe light was detected by a Si photodiode for the visible region and an InGaAs detector for the near-infrared region. Signals of transient BM and EL were simultaneously measured under the applied squared AC voltage with a frequency of 80 Hz and recorded using a digital oscilloscope (Fig. 1). The voltage dependences of the transient BM and EL signals were recorded for the top square voltage ranging from 3 V to 10 V and the 0 V-bottom voltage. The probe light for the transient BM measurements was produced from a LED. Optical filters were used in recording the BM and EL signals to block scattered EL and probe light, respectively. The optical arrangement used was carefully retained during measurements of voltage-dependence to realize accurate relative comparison. The current measurements were performed simultaneously with the transient BM and EL measurements. The steady state current were then measured by gating a saturated current with a boxcar integrator. All measurements were performed at room temperature under vacuum conditions.

## Additional Information

**How to cite this article**: Takahashi, T. *et al.* Direct monitoring of bias-dependent variations in the exciton formation ratio of working organic light emitting diodes. *Sci. Rep.* **5**, 15533; doi: 10.1038/srep15533 (2015).

## Supplementary Material

Supplementary Information
