# Supplementary material for: Direct monitoring of bias-dependent variations in the exciton formation ratio of working organic light emitting diodes
Source: Sci Rep. 2015 Oct 21;5:15533. doi: 10.1038/srep15533 (PMC4614446; doi:10.1038/srep15533)
Supplement: Supplementary Information [file srep15533-s1.pdf]

Supplementary Information

Direct monitoring of bias-dependent variations in the exciton formation ratio of working organic light emitting diodes

Takahiro Takahashi,[1] Katsuichi Kanemoto,[1,*] Mariko Kanenobu,[1] Yuta Okawauchi,[1] Hideki Hashimoto [1,2]

[1] *Department of Physics, Osaka City University, 3-3-138 Sugimoto, Sumiyoshi-ku, Osaka 558-8585, Japan*

[2] *The OCU Advanced Research Institute for Natural Science and Technology (OCARINA), Osaka City University, Osaka 558-8585, Japan.*

[*] Corresponding author

E-mail: kkane@sci.osaka-cu.ac.jp

I. Fundamental properties of device

Figure S1 shows the current-voltage characteristic and EL-intensity vs. voltage of typical MEH-PPV LED used in this study. The inset also shows the result of calculation of the EL intensity divided by current typically obtained from the LED, corresponding to a relative efficiency of the device.

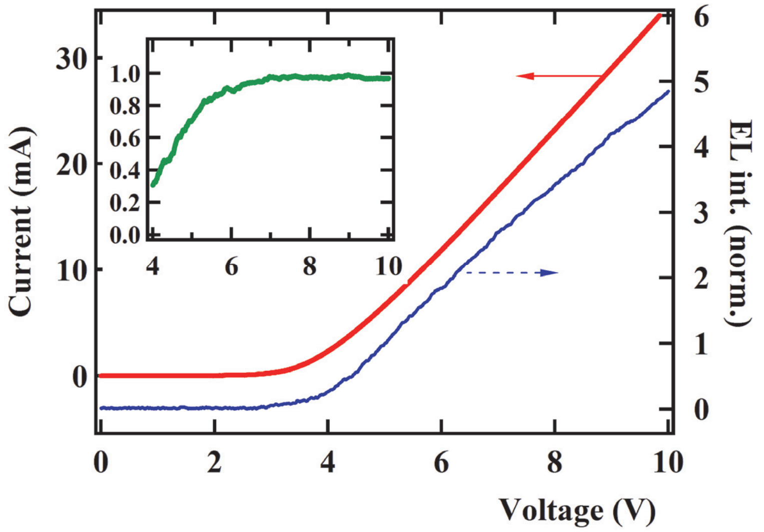

Fig. S1 Current-voltage characteristics (left axis) and EL-intensity (right axis) vs. voltage normalized at 5 V of typical MEH-PPV LED used in this study. The inset indicates the result of calculation of EL intensity divided by current typically obtained from the LED.

II. Fitting results of transient bias modulation signals

Figure S2 (a) shows the voltage dependence of relative polaron density during the LED-operation ($N_{P,\ ON}$) and after switching off ($N_{P,\ OFF}$) determined from the fits for the transient bias-modulation signals. Figure S2 (b) shows the voltage dependence of the decay time constant of the polarons during the LED-operation ($\tau_{P,\ ON}$) and after switching off the bias ($\tau_{P,\ OFF}$) determined from the same fits.

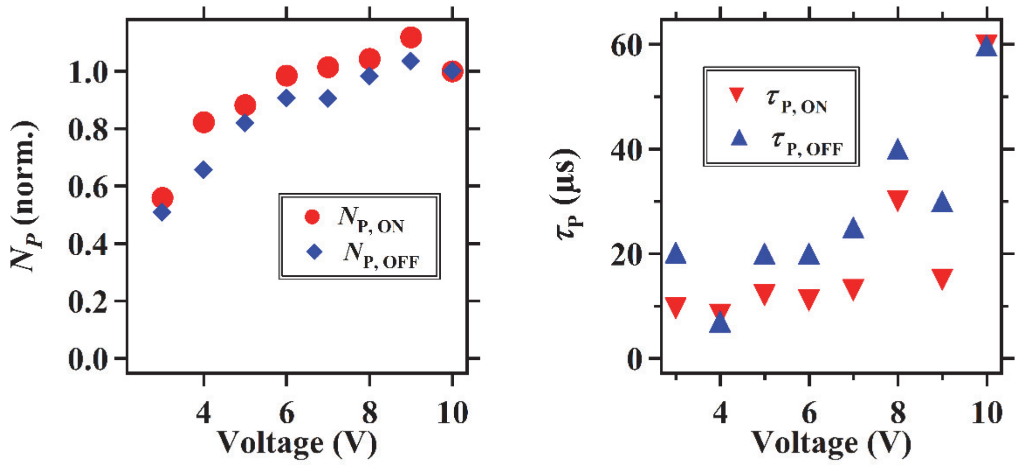

Fig. S2(a) Voltage-dependence of polaron density during LED-operation ($N_{P,\ ON}$) and after switching off the bias ($N_{P,\ OFF}$) normalized at 10 V. (b) Decay time constants of the polaron during LED-operation ($\tau_{P,\ ON}$) and after switching off the bias ($\tau_{P,\ OFF}$) vs. applied voltage.

III. Model for fitting G_T/G

A. Screening effect on the internal electric field

When applying Eq. (4) to measured G'_T/G_S, the relation between the applied voltage V and the internal field $F(V)$ in the PLED should be considered. An internal electric field within operating LEDs was previously shown to be saturated upon increasing an operation bias due to the screening effect by injected carriers [S1,S2]. For incorporating the saturation effect, we employed an exponential type function of $1-\exp[-s(V-V_b)]$ for $F(V)$, where s is the parameter of screening and V_b is the built-in voltage calculated to be 2.2 V from the difference of the work functions between Ca and PEDOT/PSS. The exponential function is particularly necessary to retain the condition of $\Delta E_S - \beta F(V) > 0$.

Figure S3 shows the voltage dependence of the normalized internal electric field ($F_{\mathrm{eff}}(V)$) used when fitting G'_T/G_S in Fig. 4(c). The internal field rises at 2.2 V corresponding to the built-in voltage and is then gradually saturated. This saturation curve is similar to the voltage-dependent curve reported previously that indicated the saturation of the internal field in operating LEDs [S1].

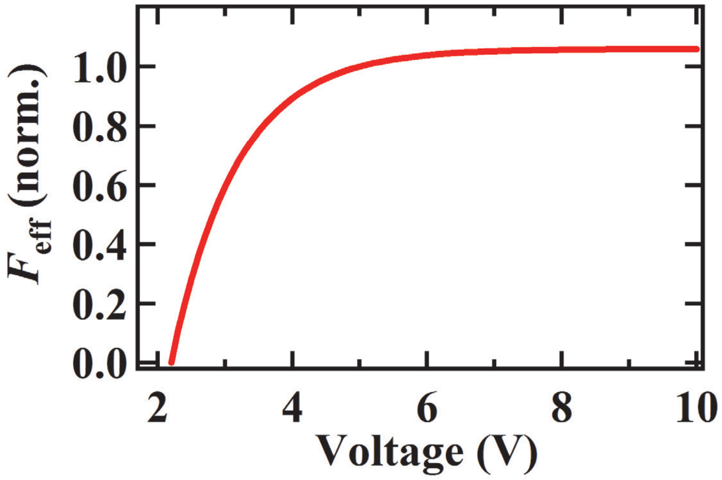

Fig. S3 Effective internal electric field F_{eff} used in fitting G_S/G_T in Fig. 4(c). The field was normalized at 5 V.

B. Estimate of the range of G_T/G_S

The range of the magnitude in G_T/G_S (or γ) can be estimated from the measured G'_T/G_S (or γ'). The result shows that γ' is reduced to about one-third by the bias-change from 5 V to 10 V. From the change of γ' and the relation of $\gamma' = \gamma + k_{\mathrm{isc}}\tau_S$, we obtain the following equation:

$$\gamma_{10V} = \frac{1}{3}(\gamma_{5V} - 2k_{isc}\tau_S), \qquad \text{(S1)}$$

where the indexes for γ represent the magnitude of applied voltages. When assuming the photoluminescence quantum efficiency to be about 0.1 [S3], the condition of $0 \leq k_{\mathrm{isc}}\tau_S \leq 0.9$ is obtained. Therefore, when using $\gamma_{5V} = 3$, the estimated range of γ is calculated as $0.4 \leq \gamma_{10V} \leq 1$. This indicate that G_T/G_S is reduced to less than one-third by the bias-change.

Reference

[S1] P. J. Brewer *et al.*, J. Appl. Phys. **99**, 114502 (2006)

[S2]C. V. Hoven, J. Peet, A. Mikhailovsky and T. Q. Nguyen, Appl. Phys. Lett. **94**, 033301 (2009)

[S3] J. C. de Mello, H. F. Wittmann and R. H. Friend, Adv. Mater. **9**, 230 (1997).
